# 2791. Antimicrobial Activity of Cefiderocol and Comparator Agents Against Isolates of *Pseudomonas aeruginosa, Acinetobacter baumannii-calcoaceticus* species complex, and *Stenotrophomonas maltophilia* by Infection Type from United States Hospitals in the SENTRY Antimicrobial Surveillance Program (2020-2022)

**DOI:** 10.1093/ofid/ofad500.2402

**Published:** 2023-11-27

**Authors:** Sean T Nguyen, Calbert Valencia, Jason J Bryowsky, Boudewijn L DeJonge, Joshua Maher, Rodrigo E Mendes, Miki Takemura, Yoshinori Yamano

**Affiliations:** Shionogi Inc., Florham Park, New Jersey; Shionogi Inc, Florham Park, New Jersey; Shionogi Inc., Florham Park, New Jersey; Shionogi Inc., Florham Park, New Jersey; JMI Laboratories, North Liberty, Iowa; JMI Laboratories, North Liberty, Iowa; Shionogi & Co., Ltd, Toyonaka, Osaka, Japan; Shionogi & Co., Ltd., Toyonaka, Osaka, Japan

## Abstract

**Background:**

Cefiderocol (CFDC) has broad *in vitro* activity against non–fermenting Gram-negative bacteria which often present treatment challenges due to multi-drug resistance. Stratified by infection type, the activity of CFDC and comparator agents was evaluated against *Pseudomonas aeruginosa* (PSA), *Acinetobacter baumannii-calcoaceticus* complex (ABC), and *Stenotrophomonas maltophilia* isolates, collected from US hospitals during 2020-2022 in the SENTRY antimicrobial surveillance program.

Antimicrobial Activity of Cefiderocol and Comparator Agents Against Isolates of Pseudomonas aeruginosa, Acinetobacter baumannii-calcoaceticus species complex, and Stenotrophomonas maltophilia by Infection Type from United States Hospitals

**Figure:**
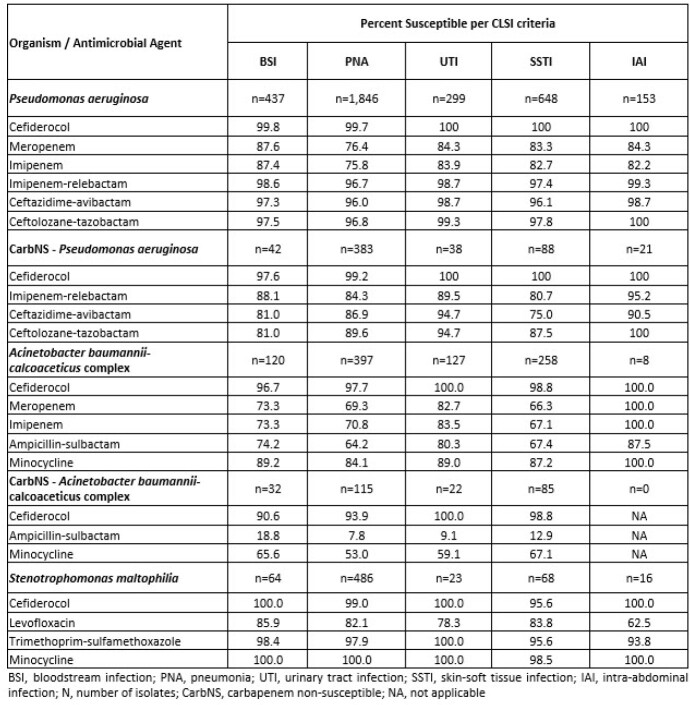

**Methods:**

3,383 PSA, 910 ABC and 657 *S. maltophilia* isolates from US hospitals were tested for susceptibility (%S) using broth microdilution with cation-adjusted Mueller-Hinton broth (CAMHB) or iron-depleted CAMHB for CFDC. Comparator agents included: ceftazidime-avibactam (CZA), ceftolozane-tazobactam (C/T), and imipenem-relebactam (I-R) as well as ampicillin/sulbactam (SAM), meropenem (MEM), imipenem (IPM), minocycline (MIN) and trimethoprim-sulfamethoxazole (SXT). Carbapenem non-susceptible (CarbNS) was defined as non-susceptibility to IPM and MEM. Susceptibility was interpreted according to 2023 CLSI breakpoints.

**Results:**

The most common infection type from which isolates were collected was pneumonia (PNA; n=2,729), followed by skin and skin structure (SSTI; n=974), bloodstream infection (BSI; n=621), urinary tract infection (UTI; n=449) and intra-abdominal infections (IAI; n=177). Among PSA, %S to CFDC was highest among comparators with >98%, across all infection types, including those caused by CarbNS isolates. In CarbNS-PSA, %S to CZA and C/T were lowest in BSI isolates while among PNA isolates, %S to CZA and I-R were the lowest. ABC %S to CFDC ranged from 97-100% and 91-100% in CarbNS-ABC across all infection types. In CarbNS-ABC, SAM and MIN %S in BSI and PNA was 19% and 66% and 8% and 53%, respectively. Against *S. maltophilia* isolates, CFDC and MIN were the most active agents across all infection types.

**Conclusion:**

Against PSA, ABC, and *S. maltophilia*, including Carb-NS, isolates from US hospitals, CFDC was the most active agent with >90% susceptibility across all infection types. CFDC represents a potential option for empiric antimicrobial therapy in US hospitals with high rates of Carb-NS non-fermenting Gram-negative pathogens.

**Disclosures:**

**Sean T. Nguyen, PharmD**, Shionogi: Employee|Shionogi, Inc: Employee **Calbert Valencia, PharmD**, Shionogi Inc: Employee **Jason J. Bryowsky, PharmD, MS**, Shionogi Inc.: Employee **Boudewijn L. DeJonge, PhD**, Shionogi Inc.: Employee **Joshua Maher, PhD**, AbbVie: Grant/Research Support|Affinity Biosensors: Grant/Research Support|AimMax Therapeutics, Inc: Grant/Research Support|Alterity Therapeutics: Grant/Research Support|Amicrobe, Inc: Grant/Research Support|Arietis Pharma: Grant/Research Support|Armata Pharmaceuticals, Inc: Grant/Research Support|Astrellas Pharma, Inc.: Grant/Research Support|Basilea Pharmaceutica AG: Grant/Research Support|Becton Dickinson And Company: Grant/Research Support|bioMerieux, Inc: Grant/Research Support|Boost Biomes: Grant/Research Support|Diamond V: Grant/Research Support|Fedora Pharmaceuticals, Inc: Grant/Research Support|Iterum Therapeutics plc: Grant/Research Support|Johnson & Johnson: Grant/Research Support|Kaleido Biosciences, Inc.: Grant/Research Support|Meiji Seika Pharma Co. Ltd.: Grant/Research Support|National Institutes of Health: Grant/Research Support|Pfizer Inc.: Grant/Research Support|Roche Holding AG: Grant/Research Support|Shionogi Inc.: Grant/Research Support|Summmit Therapeutics, Inc.: Grant/Research Support|Zoetis Inc: Grant/Research Support **Rodrigo E. Mendes, PhD**, AbbVie: Grant/Research Support|Basilea: Grant/Research Support|Cipla: Grant/Research Support|Entasis: Grant/Research Support|GSK: Grant/Research Support|Paratek: Grant/Research Support|Pfizer: Grant/Research Support|Shionogi: Grant/Research Support **Miki Takemura, n/a**, Shionogi & Co., Ltd.: Stocks/Bonds **Yoshinori Yamano, PhD**, Shionogi HQ: Employee

